# Developing an ethics support tool for dealing with dilemmas around client autonomy based on moral case deliberations

**DOI:** 10.1186/s12910-018-0335-9

**Published:** 2018-12-22

**Authors:** L. A. Hartman, S. Metselaar, A. C. Molewijk, H. M. Edelbroek, G. A. M. Widdershoven

**Affiliations:** 1 Department of Medical Humanities, VU University Medical Centre, APH, Amsterdam, The Netherlands; 2 Centre for Medical Ethics, Institute of Health and Society, Faculty of Medicine, University of Oslo, Oslo, Norway; 3 Institute for Interdisciplinary Studies, University of Amsterdam, Amsterdam, The Netherlands

**Keywords:** Moral compass, Autonomy, Clinical ethics support, Moral case deliberation, Long-term care, Moral dilemma, Theme-based ethics support

## Abstract

**Background:**

Moral Case Deliberations (MCDs) are reflective dialogues with a group of participants on their own moral dilemmas. Although MCD is successful as clinical ethics support (CES), it also has limitations. 1. Lessons learned from individual MCDs are not shared in order to be used in other contexts 2. Moral learning stays limited to the participants of the MCD; 3. MCD requires quite some organisational effort, 4. MCD deals with one individual concrete case. It does not address other, similar cases (it is case based). These limitations warrant research into complementary ways of providing CES to healthcare professionals. Our research objective was therefore to develop a low threshold CES tool based on a series of MCDs on autonomy in long-term care.

**Methods:**

We used a qualitative research design in which we analyzed the process and content of a series of MCDs, combined with reflections on the theoretical background of MCD. In total 28 MCDs (10 transcripts and 18 summary reports) were analyzed by means of a thematic content analysis. In various rounds of development, the results of the analysis were combined with theoretical reflections on CES. Consequently, the tool was evaluated in three focus groups and adjusted.

**Results:**

The CES tool, called ‘moral compass’, guides the users through a series of six subsequent questions in order to methodically reflect on their concrete moral dilemma, in the form of a booklet of 23 pages. It combines a methodical element that encourages and structures a reflection process with a substantive element, including norms, values, options, strategies, and insights regarding dealing with client autonomy.

**Conclusion:**

By using data from a series of MCDs, combined with theoretical reflections on MCD, ethics support and moral learning, we developed a thematic, low-threshold CES tool that supports healthcare professionals in daily practice in dealing with moral questions regarding client autonomy. It integrates examples and insights from earlier MCDs on the same topic. The moral compass is not a replacement of, but can be used complementary to MCD. The feasibility and impact of the moral compass need to be investigated in an evaluative follow-up study. The methodology presented in this paper may be used to develop moral compasses on different topics in various healthcare organizations.

## Background

In the Netherlands and other European countries [1–3], Moral Case Deliberation (MCD) is an established form of clinical ethics support (CES) [4]. MCD is a reflective and structured dialogue between healthcare professionals, sometimes also including other stakeholders (such as patients, family members, and volunteers) about *their* concrete moral questions regarding a real clinical case, and the underlying values and norms within a specific context [5–7]. A crucial theoretical presupposition of MCD is that the concerns of stakeholders in a concrete situation are the primary starting point for reflection, and that a moral judgement should be based on the moral experiences and reasoning of the stakeholders [5, 8]. The role of the ethicist (or trained professional) as ‘facilitator’ of MCD is to foster a dialogical reflection based on these experiences, rather than to provide normative guidance or give advice [9].

MCD is both process and outcome oriented. A specific moral question that arises from concrete experience is analysed in a structured way according to a specific conservation method. This process fosters the moral competences of the participants, reduces moral distress and contributes to mutual understanding, for instance among team members [6, 10–12]. The aim of an MCD meeting is to develop a shared perspective on ‘the right thing to do’ in a concrete situation, in which broader normative frameworks (such as law, guidelines and policy regulations) are taken into account. Evaluation studies show that MCD participants are (very) positive about MCD as a method of CES [10, 13].

Although MCD is successful, it also has limitations. First, lessons learned from individual MCDs are not shared in order to be used in other contexts. Second, MCD allows for only a limited number of people to join, learn and profit from the mutual learning process and insights generated by the joint reflection (about 8–12 per session). Third, MCD is relatively time-consuming, as sessions approximately take 90 min. Also, a trained facilitator is required [14]. Consequently, MCD comes with much organisational effort. Fourth, MCD deals with one individual concrete case. It does not address other, similar cases (it is case based). Thus, both the scope of MCD and its feasibility in practice are limited.

These limitations warrant research into a tool for CES, in line with the methodological and theoretical principles of MCD, which (1) supports both individual and groups of healthcare professionals in dealing with dilemmas; (2) integrates examples and insights from earlier MCDs on the same topic; (3) can be used by a larger number of healthcare professionals directly, without the assistance of a facilitator; (4) takes less time and less organisational effort. In order to address these questions, we aimed to develop a CES tool to provide support in cases with the same central topic, and with a lower practical and organizational threshold than MCD. Ethical-theoretical viewpoints on clinical ethics support, MCD and the CES tool have been important for the development of the compass. Due to limited space within this paper, we describe our theoretical reflections (based on hermeneutic ethics and pragmatism) for the development of a CES tool based on a series of MCDs in more detail elsewhere; here we focus on the actual development of the tool [8].

In order to develop a tool for CES complementary to MCD, we used results from a series of MCDs with healthcare professionals. Based on a qualitative analysis of the process and the content of the series of MCDs, as well as reflections on the theoretical background of MCD, ethics support and moral learning, we developed a so-called ‘moral compass’, in the form of a booklet of 23 pages. We use the term ‘moral compass’ to designate an actual materialized tool that guides the user(s) through a reflective process by means of offering both a structure for the reflection and substantial elements regarding the moral issue at stake. This is to be distinguished from the way in which this term is used in psychological literature, referring to one’s inner, psychological moral framework [15]. In developing the moral compass, we focused on moral questions concerning client autonomy in long-term care contexts.

Within Dutch healthcare, there is an increasing emphasis on ‘demand-driven care’, ‘self-determination’ and ‘client centeredness’, three concepts which all stress that clients should be enabled to live their lives as autonomously as possible, and that their individual wishes, demands and needs should be central to the process of care. In this context, the empowerment of clients through a redistribution of responsibilities and control among healthcare professionals and patients is encouraged [16]. However, this focus on autonomy gives rise to ethical issues, especially for caregivers whose clients have cognitive impairments (mental disability, dementia), or a psychiatric disorder. Often, these clients need long-term care services, including personal care and coaching in life skills, such as handling money, personal hygiene, relationships and jobs. Caregivers providing these services are regularly confronted with situations in which they experience a conflict between providing good care on the one hand and going along with the wishes and preferences of their clients at the other hand. The central moral question for many healthcare professionals therefore usually goes like: “When should I respect the client’s autonomy and expressed needs and when should I overrule them either to prevent harm or to do well?”

The overall aim of this study was to offer a tailor-made and easy accessible CES tool, transferring insights from a series of MCDs regarding a specific theme to other healthcare professionals in similar situations, thereby contributing to a more efficient way of moral learning throughout the whole organization. As such, this CES tool can be part of an innovative, practical way of offering CES; moving from a case-based CES to a theme-based CES.

In the following, we first describe the methods we used for the development of this moral compass. After presenting an outline of the moral compass, we describe specific aspects of both the methodical structure and substantial content of the moral compass. In the discussion, we go into the similarities and differences between the moral compass and MCD, and address strong and weak points of the moral compass as an innovative CES tool.

## Methods

### Research setting and research team

This research project was performed in a large healthcare institution, which provides predominantly long-term care to more than 19.000 clients in the Netherlands, and has more than 8.500 staff members. MCDs were organized in three divisions of this institution: Nursing and Caring, Care for people with a mental disability, and Care for psychiatric patients. The first division has twenty-six locations, consisting of nursing homes, residential homes and small living homes. Care for the mentally disabled consists of more than fifty locations for individuals as well as group homes. The division for psychiatric care has eleven locations for assisted living facilities, daily activities and several working facilities.

In each of these divisions, a series of MCDs was organized. During the MCDs, professionals presented actual moral dilemmas they struggled with. Frequently, these dilemmas concerned the choice between either respecting client autonomy by going along with a client’s wishes versus active intervention, sometimes even against the client’s wish. This topic had also been mentioned as one of the most important causes for moral doubt in an exploratory interview study with 14 employees, next to issues related to cultural differences, and dealing with misconduct by both colleagues and clients. Based on the findings on the interview study, we decided to focus the content of our moral compass on moral issues concerning client autonomy.

The first author, second author and fourth author (as a participating intern) assembled and analyzed the data from the MCDs. The third and fifth author participated in the last steps in the analysis process. All authors contributed to the theoretical reflections on MCD, ethics support and moral learning. The first author and fourth author organized the focus groups in which the tool was evaluated. All authors were involved in constructing the final version of the tool.

### Research design

The research had the specific aim of developing a moral compass to support the future user in dealing with concrete moral dilemmas regarding client autonomy. As such, the CES tool was not merely descriptive (i.e. limited to describing the kind of moral dilemmas healthcare professionals experience), but also user- and action oriented. The research design was inspired by a responsive evaluation approach. Responsive evaluation seeks to give stakeholders a voice [17, 18], and actively involve stakeholders such as professionals, patients and family members to contribute to research agenda’s, the research itself, the evaluation of practices and the improvement of practices [19, 20]. For this research, we involved the participants of the MCDs as stakeholders.

The research design combined empirical research and theoretical reflection [21]. During the content analysis of the data from the series of MCDs, and during our regular research group meetings, we theoretically reflected upon the structure and the content of the moral compass we aimed to develop. What should be the implications of the theoretical background of MCD [22] for the actual form(at) of the moral compass and the theoretical aims of the steps within the format? These theoretical reflections influenced the way we used the data in designing the moral compass.

Examples of these theoretical reflections were: the moral compass should stimulate moral reflection and not dictate what had to be done; the moral reflection should be based on an actually experienced moral dilemma; the moral compass should encourage moral learning of users by making explicit how they think themselves and how others might think about the same issue (e.g. by incorporating other perspectives, different values and other content). In another paper on the theory of the moral compass, we will reflect further on the rationale and theoretical background of the reflections which were part of the developmental process of the moral compass (forthcoming).

### Data collection

To obtain insight into moral dilemmas experienced by professionals, as well as the actual reflection and dialogue between professionals on these dilemmas on client autonomy, the content of a series of MCDs was analyzed. The MCDs used a structured conversation method; the dilemma method [5, 7]. Within each division of the healthcare institution, a series of monthly MCD meetings was organized. The meetings took place between January 2013 and March 2014 at various locations of the healthcare organization. During these meetings, which lasted about 2 hours each, participants discussed and analyzed a moral dilemma they experienced themselves in daily practice by using the dilemma method. Participants were asked before the meeting whether there was a situation in the case of which they doubted what was ‘the right thing to do’. This request was formulated in an open way, in order to avoid the influence of the preconceptions of the researcher on the characteristics of a moral dilemma. When more than one moral dilemma was mentioned, the group democratically chose the dilemma they wanted to analyze themselves. Thus, the theme of the MCDs was not determined in advance.

The MCDs were semi-closed. This means that a core group of participants attended all MCDs, and that for each specific MCD other professionals were invited, for example when a professional from outside the regular group wanted to present a moral dilemma, often accompanied by one or more colleagues involved in the case. The MCDs were multidisciplinary (participants regularly included several daily caretakers, a physician, a manager, a client representative and/or a former client or relative with experiential expertise). Due to incompatible work schedules or sickness not every participant attended every meeting.

The conversations during the MCDs were audio-recorded. These recordings were used to make a summary report of the meeting that was send back to the participants for a member check. Besides the reports, 10 MCDs were transcribed ad verbatim. 24 of the 28 MCDs were facilitated by one of the members of the research team, the other four were facilitated by another trained MCD facilitator [14].

### Data analysis

Through a thematic content analysis, we gained an in-depth understanding of the dialogues within the MCDs [23]. During the analysis we mainly focused on the following three lines of inquiry; 1) ‘How do the MCD participants describe the moral tension between actively intervening in a situation or respecting the choice of a client?; 2) ‘On which values do the MCD participants base their choices?’, and 3) ‘What kind of actions follow from these values/circumstances?’

We made a list of all MCD meetings and read the reports, to familiarize ourselves with the data. Two transcripts were selected to prepare the coding process. These were transcripts in which the theme of the moral compass, i.e. client autonomy, has been explicitly discussed. The first, second and fourth author read these two transcripts separately and coded the transcript line for line, categorizing the data according to the three questions mentioned above. Next, they discussed the coding and sought consensus over diverging interpretation of the data. The aim was to establish a shared coding system for the following phases of analysis. After this, eight more transcripts were coded independently, one by the first author, one by the second author and six by the fourth author. Subsequently, the remaining 18 summary reports were read by the first second and fourth author, in order to check whether there was information in the reports that we had not found within the transcripts. We found that 25 of the 28 MCDs discussed client autonomy in one way or another. We reached data saturation, as we did not find new information related to the theme of client autonomy and were able to fit all content of the 18 reports of the remaining MCDs in the established structure based on the 10 transcribed MCDs. The findings were discussed with all authors.

### Development of the tool

The analysis of the data provided insight into a) what dilemmas related to client autonomy professionals experience, b) which values are important for them when being confronted with these dilemmas, and c) which actions follow from the reflections upon these dilemmas. These outcomes were used as input for the development of the moral compass. As the moral compass was intended to foster reflection, a series of questions was developed, inciting the user to make explicit his or her dilemma, relevant values and possible actions. The results of the data analysis helped to formulate these questions and to illustrate them with examples and insights from earlier MCDs. The examples entailed (a) a case example which was paradigmatic for the kind of cases the professionals brought to the MCD with concern to client autonomy. In each step of the compass, an answer was given to the central moral question on the basis of the reflection on this case. In this way, the moral compass guides the user in which *kind* of answer was expected (in a formal sense). The examples also entailed (b) values and norms that came up frequently as important in the MCDs; and (c) ways of dealing with dilemmas concerning client autonomy that the MCD participants often came up with. The process of developing the moral compass entailed various steps of reflection, in which the researchers discussed how to relate the steps in the tool to the theoretical presuppositions of MCD.

#### Try-out and evaluation

Subsequently, one focus group was organized in each division of the organization (i.e. three in total), in which we presented a first draft of the moral compass to the participants of the MCDs (May/ June 2014). The focus groups were facilitated by the first and fourth author. In the focus group we asked the participants to imagine a recent moral dilemma and use the moral compass to reflect on that dilemma. During the try-out we asked them to reflect on their experiences with the moral compass and to write down evaluative comments on the compass. We collected, discussed and organized the comments in three categories; elements considered useful, elements which needed improvement and ‘other’ (anything that did not fit the preceding categories). We encouraged the participants to discuss diverging views on the moral compass in the meeting and come to a consensus. We made a report of all the comments and send it back to the participants. The first, second and fourth author considered independently which comments to incorporate in the second draft and met to discuss their findings, compared views and discussed diverging views on which comment to process in which way. Then the second draft was discussed with all five authors.

A graphic designer was approached at an early stage, not only to make the moral compass user friendly, visually attractive and accessible, but also to come to a design that instigates a well-structured reflection process.

##### Quality measures

In the process of analyzing we continuously sought consensus and discussed diverging views research group to avoid bias (researcher triangulation). Also, we organised various member checks during the research process 1) there was a report of every meeting that has been sent back to the participants, 2) we organized three focus groups on a first draft and 3) we sent a report of the focus groups back to the participants to check whether we had understood the comments correctly. We did not preselect only those MCDs that focused on the theme of the moral compass, but took all MCDs into consideration, to avoid bias.

##### Research ethics

We asked for explicit permission to record and transcribe the MCDs and use them for analyses. At every MCD the confidentiality of the meeting was emphasized and we made sure that no content in any public document can be led back to individual persons. For this reason we changed some details in the description of the moral dilemmas in this paper. Since the research does not include patients in a direct way, no permission was needed from the Medical Ethics Review Committee according to the Dutch Medical Research on Human Subjects Act (WMO) [24].

## Results

The moral compass we developed has two components. 1. A methodic component: six subsequent questions that encourage structured reflection and dialogue concerning a moral dilemma 2. A substantive component: norms, values, options, strategies, best practices, insights regarding client autonomy based on the qualitative analysis of the series of MCDs. In the following, we first present an outline of the moral compass, after which we go into specific aspects of its methodical structure and substantial content, by describing eight main characteristics of the moral compass.

### Outline of the moral compass

The moral compass has the form of a booklet, with several questions, and space to write answers to encourage the user to reflect on their moral dilemma. We also developed a digital version in which the answers can be typed.

The moral compass contains a series of six questions (Q1 to Q6). The user of the compass is asked to answer the questions for their own moral dilemma. See Table 1 for an overview of the questions. Each question is accompanied by an explanatory sentence about why answering this question may be helpful for the moral dilemma at hand (aimed at the understanding of the process by the user) and why answering this question may be helpful for the user of the moral compass.

**Table 1:** Six questions and their explanations from the moral compass (translated from Dutch)

Question Number	Questions and their explanations from the moral compass
Title Page	*Do I follow the wish of the client or do I intervene? First aid with daily dilemmas. Moral compass*
Q1	*Sometimes, it is not self-evident what is the right thing to do.* What is your dilemma?
Q2	*It may be helpful to clarify what exactly makes the situation difficult*. What causes you to have moral doubts in this situation?
Q3	*By placing yourself into the shoes of others, you acquire valuable insight into different perspectives on the problem and on the right thing to do. With each question, ask yourself what would be important to someone, both in the long and short run.* What is important to whom? Q3A: Place yourself in the shoes of the client: what is important for the client? Q3B: What is important for you? Q3C: Place yourself in the shoes of the others who are involved in the situation; what is important for them? (colleagues, network, other clients, management?) Q3D: What do rules and regulations say about this situation?
Q4	Think about what is important for all the parties involved (as answered for Q3). Please make a consideration in which you take all perspectives into account. Q4A What is most valuable to you in this situation? Q4B: Which actions go along with this?
Q5	*Often, there is no perfect solution for a dilemma. Each choice has its disadvantages, because you simply cannot do everything that is important. It helps to be conscious of this, so you can possibly limit these disadvantages.* Q5A: What is a (possible) disadvantage related to your chosen course of action? Q5B: Can you do anything to compensate for this disadvantage?
Q6	Are you able to deal with your dilemma after using this compass?

The six questions are illustrated with content based on the analysis of the MCDs. This content consists of, for instance, examples of the issues participants from the MCDs struggled with and best practices the participants of the MCDs identified. Also, an exemplary case is presented, about a client who does not want to make any lifestyle changes even though at great health risks (a common moral dilemma within the theme client autonomy). All six questions are elaborated for this exemplary case, to provide the user with some guidance regarding *what kind of* answer is expected. This is only for heuristic purposes: it is explicitely stated that the exemplary case in no way is meant to provide an answer to the moral dilemma of the user.

### Characteristics of the moral compass

The following eight characteristics of the moral compass aims to encourage reflection by the user(s) of the moral compass.

#### Providing support by combining an exemplary case with substantive content

To support caregivers with their moral dilemmas, we developed a structure that encouraged reflection not in an abstract way on an abstract dilemma, but rather, reflection on *their own*, concrete moral dilemmas as these are experienced in daily care practices. We used a simplification of the steps of the dilemma method as a basic structure for the whole compass. This resulted in six questions (see Table 1), inviting the user to focus on his or her own experience. A concretely experienced case is of crucial importance when moral learning is an objective (which is an aim of this CES tool). Furthermore, the details of the situation determine what is morally relevant and what is morally right.

We added an exemplary case for which all six questions are answered. The use of an exemplary case was suggested in the focus groups, which showed that users struggled with answering the questions of the moral compass for their own moral dilemma.

Besides the questions and the exemplary case, we added substantive content (see below for a specification of this content). The added content has two functions; 1. It provides the user with ideas and suggestions that may be helpful for understanding their moral dilemma. The suggestions are exemplary in the sense that when these types of moral dilemmas occur, often these specific values are mentioned when discussing moral issues concerning this theme. The content case provides possible action strategies and services that are available within this specific healthcare organization for providing care (i.e. it is contextualized). 2. It frames the type of answers that can be given to the questions and the way they could be formulated by the user.

#### Putting oneself in the position of the client

We asked the following question in the moral compass: ‘Put yourself in the position of the client; what is important for the client?’ We did so because the analysis showed that putting oneself in the place of the client often provided deepened and broadened understanding of the moral issue at hand and of what should be done. The participants stated a difference between thinking *about* the client and determining what is best for him or her, and putting oneself actually in the shoes of the client and then asking: ‘What would be important for me, if I was in this situation?’ We found that this exercise could have different consequences. In some MCDs it provided the participants with more insight into themselves and their own values and norms. For instance, a participant remarked: ‘I think safety is important, but if I would have two supervisors present at my first date, I would hate that’. And ‘If it were me in this situation, I would start protesting immediately, but is this the same for everyone?’ At other times this question encouraged participants to actively consider all values that could be important for the client, for instance that different values were probably important for the client, but the situation did not permit to honor all values. In some cases, the MCD participants came to realize that there was too little information on the values of the client and that more time should be invested to ask the client his or her perspective. Finally, it was sometimes acknowledged that the care giver could not realize the expressed wishes of the client. However, the care giver became aware that he/she was able to honor *the value* behind the expressed wishes, by doing something else, which was considered a preferable option.

#### Finding a middle ground and going beyond the dilemma

Based on the analysis of the MCDs, the theoretical reflections and the focus groups we found that ‘solving’ the dilemma and finding action strategies was usually not a matter of weighing arguments for A and B, nor choosing for action A or action B of the dilemma. Through reflections on the different meanings of the underlying values associated with the two sides of the dilemma, and on how participants think that values can be realized through certain actions, participants reconstruct a moral answer to the initial dilemma. For example, in the final consideration participants of the MCD often did not choose one action over another but actively tried to find a middle ground beyond for instance respecting the wishes of the client and trying to secure the client’s safety or health. Sometimes, due to the deliberation, the original dilemma changed or disappeared since the presuppositions on which the dilemma was built changed during the ethics reflection. At other times participants still chose one side of the dilemma, but creatively limited the harm of the choice, doing justice to the other side of the dilemma or finding an intermediate solution. In the MCDs, they often discovered and shared a lot of action strategies that enabled the care giver to do this. We also incorporated these action strategies in the moral compass. For instance, ‘consider including a third party in the situation that can play the role of the intervening partner’ (when an intervention was deemed morally justified), in order to continue a relationship of trust with the client (a so-called ‘good cop, bad cop’ strategy). Or (when an intervention was not deemed morally justified) ‘invest time and use creative strategies to make the client aware of the long term consequences of for instance his or her lifestyle (for instance by showing pictures)’.

Other action strategies that were suggested in the MCDs and incorporated in the moral compass were: ‘involve third parties such as an emergency service or experiential experts’, ‘continuously discuss the situation with the client’, ‘report the situation to a regulatory agency’, ‘give the client room to make mistakes’, ‘consult the team manager’, ‘discuss the situation with the family’, ‘do nothing further and accept the consequences’, and ‘have patience when the situation cannot be solved immediately’.

#### Acknowledging rules and regulations (and their underlying values)

We found in the analysis of the MCDs that rules and regulations were regarded as relevant, but that knowledge of rules and regulations often did not solve the moral dilemma. Some participants expressed that existing rules and regulations should not always be followed automatically. It can be morally justified to make a well-argued and documented exception to an existing rule. It was also mentioned that the rule can be the source of a moral problem; e.g. the rule prescribes to do X, but it in this situation one does not think this is justified. Often, it was unclear how the rules applied to a certain situation. The analyses of the MCDs showed that it can be insightful to ‘be reminded’ of the values behind the rules, since rules are sometimes experienced as bureaucratic and limiting. Rules and regulations are often inspired by values (for instance respecting the autonomy of people who are in a vulnerable situation) that are sometimes ‘out of sight’ within the care context. In the moral compass, we asked the question: “What do rules and guidelines say about this situation”. In the example, we incorporated elements of the Dutch Law on restraining measures for clients within long-term care.

#### Offering examples of various moral dilemmas helps to apply the moral compass to the moral dilemma of the user

Sometimes participants of the MCD stated that they experienced a specific situation as a *morally* difficult situation *only after the MCD*, whereas before they had interpreted the situation merely as difficult. This finding implies that in not always immediately clear for caregivers, for what kind of issues the moral compass is suitable and can offer support. Besides explaining this at the beginning of the moral compass, we added various examples of the kinds of dilemmas the moral compass is suitable for. The sentence ‘The client wishes to live independently, but we do not think this is a good idea’, was incorporated as a contextual example of an instance in which a client wishes to do something of which (a team) care giver(s) do not think it is good idea. Also, we incorporated the questions; ‘Do I follow the wish of the client or do I actively intervene?’ and ‘Do I engage in a discussion about this issue with the client myself or do I report this situation for instance, to family members?’.

In the focus groups we found that the intermediary role of caregivers between the client and the family often complicates an already difficult situation with a client, or may even be the course of the moral dilemma (i.e. ‘The family wants me to do X, but I do not think this is the right thing to do for the client’, or: ‘The client wants me to do X, whereas his daughter wants me to do Y’). We therefore stimulated reflection on the role of the family by adding the exemplary question in the moral compass: ‘To what extent should I take the wishes of the family into account?’

#### Sharing responsibilities and acknowledging the tragic character of dilemmas

A dilemma entails a tragic choice. Whatever you do in the face of a moral dilemma, there will be harm; hence the designation of a moral dilemma as a ‘choice between two evils’. We found that this insight may provide a relief for caregivers, since caregivers often keep searching for the right solution, that is, the action that does no harm. The insight that harm is unavoidable, makes it easier to reflect on the harms on both sides and to explore whether it is possible to limit the harm (this is also an explicit step of the dilemma method). This insight is both theoretically inspired by the presuppositions underlying the dilemma method and writings of philosophers, such as Martha Nussbaum [25]. The insight is also confirmed by the empirical analysis of the processes within the MCDs and focus groups.

We incorporated this insight in the moral compass by asking the user of the moral compass: “What are possible disadvantages of your consideration?” We also used examples of harms that were mentioned by the participants of the MCDs; for instance: ‘the safety of others is at risk’, ‘the health of the client is at risk’, ‘you act against the wishes of the client’, and ‘the relationship with the family may deteriorate’. Furthermore, we incorporated examples of actions that may limit harm. For instance, to choose the strategy to ‘share the responsibility with others’. Some participants realized during the MCD meetings that they were not solely responsible for all aspects of the life of the client, where they initially felt solely responsible. We made this insight accessible for the users of the moral compass by adding the question; ‘Where does my responsibility for the wellbeing of the client end?’. We incorporated the suggestions ‘share your responsibilities with colleagues’ and ‘discuss the case with your team manager’, as suggestions which provided support according to the analysis of the MCDs.

#### Distinguishing long-term and short-term consequences

A helpful distinction resulting from the analysis of the MCDs was the difference between the consequences of a choice on the long and short term. In some cases, deliberation on both the short term and the long term consequences of a decision resulted in the insight that a certain course of action would lead to an endless struggle with the client that would not stop and would not be beneficial for the client (although the current situation was not ideal either). In other cases, considering the long-term consequences showed that the choices of the client would become more restricted on the long term then they would be now when one intervened. The consideration of the long and short-term consequences proved to be an important dimension to take into account in moral issues in long term care. We did not define “long” and “short” in the moral compass, since these terms may mean different time frames depending on the user and moral dilemma the moral compass is used for. We incorporated the distinction between short-term and long-term consequences in the moral compass, by asking in the inventory of values form the different perspectives: ‘ask yourself in each case what would be important on both the short term and the long term.’

#### Offering a natural terminology to discuss moral elements

During the process of development and the feedback the use of the right terminology turned out to be crucial. Through the analysis of the MCDs and the focus groups about the use of the moral compass we gained insight into how caregivers voice or verbalize certain moral themes and with what kind of ethics terminology they struggle. For example the phrase: ‘Should I put myself above the client, or ‘besides’ the client’ was often uttered and well understood by the participants of the MCD. We deliberately avoided concepts like ‘value’, ‘moral problem’ or ‘harm’ since our analysis showed that the participants often struggled with these concepts and became discouraged and distracted when asked; ‘What are the relevant values in this situation?’ or ‘Please, define the moral problem of your situation’. Instead, we used the phrase; ‘What would be important for X [a certain stakeholder’]?’ and included a list of possible values as answers, like ‘wellbeing, protection, autonomy, freedom of choice, safety, health etc.’ and instead of using the term ‘moral’ we used the phrase ‘the right thing to do’ and instead of ‘harm’ we used ‘disadvantage’. In this way, we indirectly informed the participants about values or moral questions, without providing abstract instructions which might distract or discourage users of the moral compass.

## Discussion

In this paper, we described the development of a moral compass based on the qualitative analysis of the content and the process of a series of MCDs, thereby including theoretical reflections on MCD, ethics support and moral learning. In addition, we tested the compass in three focus groups. The moral compass is intended to be a complementary tool to MCD (i.e. not to replace MCD). The moral compass is a low threshold instrument, enabling reflection in daily practice and transferring moral insights from a series of MCDs to other professionals within the organization. As such, the moral compass is an example of going beyond a case based CES to a theme based CES.

The moral compass provides no answers or moral judgments with respect to the right thing to do in the concrete situation at hand. It presents questions to stimulate refection on a moral dilemma, and suggests possible values, norms, action strategies and insights that participants of the MCDs found particularly helpful. As such, the moral compass aims at stimulating moral learning, by encouraging users to reflect upon different perspectives and to consider and weigh several relevant values and norms. This is in line with other approaches to ethics education that focus on (moral) learning and developing moral competence instead of being rule or compliance based [26–30]. The moral compass was specifically designed for one particular health care organization and on one particular moral theme (client autonomy). It requires further research to what extent the moral compass can also be used in other health care organizations for moral dilemmas surrounding client autonomy. This context specificity to a particular clinical context is also the case for another recently developed CES ‘intervention’ ETHICO [31]. We do not think this moral compass is suitable for any other moral themes then ‘client autonomy’, other moral themes require the development of other CES tools. Also, we developed a poster to inform users of the kind of questions the moral compass is suitable for, to inform users.

The moral compass shows various similarities and differences with MCD in which the dilemma method is used (see Table 2). In line with the dilemma method, the focus is on a moral dilemma, the harms of both sides of the dilemma, and ways to limit these harms. Further similarities are the focus on different perspectives and norms and values, and the importance of placing oneself in the shoes of the client. Different aspects, that are not part of the steps of dilemma method, are the distinction between long- and short-term consequences and the concrete reference to existing rules and guidelines, including the suggestion to reflect on the values behind them. This reflection on rules in line with literature which argues that knowledge of and refection on rules and regulations are mutually supportive in CES [32, 33]. Another difference is that the moral compass was designed to offer CES regarding one moral theme, i.e. client autonomy. MCDs are open and offer support on any moral issue.

**Table 2:** Comparison of the moral compass and MCD

	Moral compass	MCD
Aims	Individual reflection on morally problematic issue	Joint reflection and dialogue, possibly consensus
	Theme based	Case based
Focus	Clarification and structure	Besides clarification & structure MCD also group dialogue, openness, exchange of viewpoints
	Moral learning by, different perspectives, elucidation, encouraging structured moral reasoning	Moral learning based on a structured dialogue, supported by a facilitator
Use	Designed for use in 15–30 min	90–120 min
	To be used at any moment and anywhere	Need for a set date and time
	Independent use (individual or in small groups)	Need for a facilitator
Content	Encompasses content of colleagues	No substantive element

A crucial difference between the moral compass and MCD is that the moral compass can be used by an individual or by small groups without a facilitator, whereas MCD involves a meeting of a larger group of persons with a facilitator. So, the moral compass can both be used by one individual as well as by a small group, for instance, to structure the conversation or prepare for a team meeting. The possibility of individual use is both a strong point and weak point of the moral compass. Individual use is strong, as using the moral compass takes less time and less organization, but also weak as the process of reflection is not stimulated by concrete others and the MCD facilitator. We tried to compensate this by inserting information from other perspectives (based on the MCDs analysis). Furthermore, at the end of the moral compass, the option of additional interprofessional reflection is mentioned in case questions are left open. Thus, the user is encouraged to request for a regular MCD or to discuss the issue with a colleague, the team manager or during a team meeting.

A further difference between the moral compass and a regular MCD is that in the moral compass the substantive content is already given, whereas in MCD it depends on the contributions of the participants which content is raised. This may be a regarded as limitation of the moral compass, since the possibility of providing totally new and directly relevant values and perspectives is absent. It can also be seen as an advantage since the moral compass explicitly invites the user of the moral compass to take many aspects into account, whereas in an MCD this depends on the openness of the participants and the skills of the facilitator.

### Strengths and limitations of the study

A strength of the study is the combination of empirical research and theoretical reflection. This is in line with arguments in the literature for making explicit the theoretical viewpoint on CES in studies on the quality of a CES instrument [34, 35]. Such studies should not only focus on the practical value (usefulness and acceptability) of CES- methods, leaving theoretical reflection for the discussion section, but provide information on theoretical presuppositions from the start, using these in the design of the CES tool. By reflecting on the theoretical background of MCD, ethics support and moral learning, we were able to consciously employ theory in determining the research strategy and the process of development of the moral compass [8].

Another strength of this research project is the collaboration with caregivers from the healthcare organization. The moral compass was developed on request of, together with and for caregivers. This means that the ethics support tool has a high chance of actually supporting caregivers with their moral problems within care practices. As such the moral compass can be interpreted as part of an integrative approach of CES, i.e. an approach in which both the development and the specific use of CES is closely connected with the local circumstances and the involved stakeholders.

There are some important limitations of this study. First, the content of the moral compass is based on the participants of the MCDs. Although, this included patient representatives and experience experts, we regard it as a limitation of this research that the voice of the patient itself is missing [36]. Second, the actual use of, and experience with using the moral compass requires further research. What is the impact of the use of the moral compass on the care for clients? Does the moral compass actually stimulate reflection and moral learning? What actually happens when users of the moral compass apply it to their own moral dilemma? How do professionals prefer to use the moral compass; i.e. individually or in small groups? Do individual users of the moral compass learn something new from the suggestions, values and viewpoints incorporated in the moral compass, or does the moral compass merely lead to confirmation of their original point of view? Do they experience the example as instructive, or maybe as too directive? In sum, what kind of moral learning takes place when caregivers use the moral compass without a dialogue with other colleagues and without the instructions of a MCD facilitator?

## Conclusion

This paper presents a tool for ethics support in long-term care, focusing on moral dilemmas concerning client autonomy. This tool, called ‘moral compass’, is complementary to MCD as a method in CES. It is a low-threshold instrument, which can be used independently by individual professionals or in small groups in the context of daily care practices. The moral compass was developed on the basis of the analysis of a series of MCDs, combining qualitative empirical research and theoretical reflections. Subsequently, the moral compass has been refined through an evaluation of a try out in three focus groups. It provides a structured set of questions, and provides substantive content in order to encourage reflection and moral learning. With this research project we were able to make the insights of a series of MCDs more widely accessible in a healthcare organization. This method can also be applied to develop moral compasses on other topics.

## Abbreviations

CES: Clinical ethics support
MCD: Moral case deliberation
Qx: Question number
